# Ensuring data integrity of healthcare information in the era of digital health

**DOI:** 10.1049/htl2.12008

**Published:** 2021-04-16

**Authors:** Mohammad Zarour, Mamdouh Alenezi, Md Tarique Jamal Ansari, Abhishek Kumar Pandey, Masood Ahmad, Alka Agrawal, Rajeev Kumar, Raees Ahmad Khan

**Affiliations:** ^1^ College of Computer & Information Sciences Prince Sultan University Riyadh Kingdom of Saudi Arabia; ^2^ Department of Computer Application Integral University Lucknow Uttar Pradesh India; ^3^ Department of Information Technology Babasaheb Bhimrao Ambedkar University Lucknow Uttar Pradesh India; ^4^ Department of Computer Applications Shri Ramswaroop Memorial University Barabanki Uttar Pradesh India

## Abstract

Data integrity continues to be a persistent problem in the current healthcare sector. It ensures that the data is correct and has not even in any manner been improperly changed. Incorrect data might become significant health threats for patients and a big responsibility for clinicians, resulting in problems such as scam, misconduct, inadequate treatment and data theft. This sort of endangering scenario causes tremendous difficulty in handling healthcare data. This research intends to describe the threat plot of data integrity in healthcare through numerous attack statistics from around the world and Saudi Arabia and identify the criticality in Saudi Arabia in particular. A literature review by descriptive analysis, unit analysis and rating analysis to achieve the planned systematic literature review goal is outlined. The outcome of ranking analysis using a fuzzy analytical hierarchy process methodology offers a route for Saudi Arabian researchers to promote medical records or data security in Arabic healthcare. It is suggested that blockchain is the most prioritized method for regular use and adaptation across Saudi Arabia in all data integrity management techniques. To address the challenges of data integrity and future path, the authors critically examine the challenges posed by data integrity in the healthcare sector.

## 1 INTRODUCTION

The massive growth in the development and integration of emerging technologies in practically every part of our lives and society tends to create incredible possibilities; however, it also generates specific challenges. Data Integrity is the most sensitive concern for the current healthcare industry. Data integrity describes the way of ensuring data quality, efficiency and continuity throughout its life cycle. In the healthcare sector, it can include keeping patient's private information, health report, diagnostic reports, laboratory tests reports and other records. Data integrity management is a difficult task for health professionals and research scientists. Attackers specifically target healthcare sub‐domains to manipulate valuable data. Hence, protecting the integrity of data in medical is the most prioritized issue. For better experience and fewer infrastructure requirements, each country is pursuing to be a digitized healthcare sector. However, the process of digitization towards the healthcare sector poses many complicated challenges for security experts. Attacks on confidentiality, privacy violations, information breach credibility and many other risks are constantly growing problems for procedures and experts in digitalization. Healthcare provision is the most essential focus area for the Kingdom of Saudi Arabia (KSA) in all these emerging issues. Vision 2030 aims to lead the Kingdom as a Middle East Asian leader and a country of innovation and a prosperous economy [1]. KSA's mission and vision would establish a need for a secure data system for healthcare in the Kingdom.

Besides, maintaining data integrity is a more critical problem than other KSA cyber threats, according to a report. Tampering with health records and information about healthcare can cause a life‐threatening situation for any patient. The objective of our study is to investigate the different data integrity management strategies used by high‐quartile published papers worldwide and then identify the high‐priority technique to research the challenges and consequences of that particular methodology in the Saudi Arabia healthcare industry. The proposed research will concentrate on different approaches to data integrity used by researchers and will examine the complexities of prioritized approach to promoting the Saudi Arabian healthcare sector from the perspective of KSA.

The remaining parts of our studies are structured as: The first section discusses the critical threat plot of data integrity in the current healthcare sector worldwide and the KSA's perspective. Through the first section the reader gets to know about the recent critical and challenging situation of data integrity breaches as well as the actual situation of the Saudi Arabian healthcare sector. After that, the authors conduct a systematic literature review (SLR) with various analysis approaches. Descriptive analysis of literature provides information about previous trends and techniques of data integrity in healthcare, and unit analysis presents a clear view of prior techniques used to facilitate only a subpart of healthcare infrastructure. Ranking analysis provides a prioritization technique for various data integrity techniques and selects a most prioritized blockchain approach for KSA's healthcare sector as a suggestion for future research. After the evaluation of techniques through the Fuzzy analytical hierarchy process (AHP), the authors portray the challenges and future directions of the topic. In the concluding marks, the authors discuss the results of the study and conclude the paper.

## 2 CURRENT TRENDS IN DATA INTEGRITY RISK

Managing data integrity is a crucial task for experts in healthcare. Various challenges associated with information management in healthcare create many possibilities for attackers to exploit the organization [2, 3]. But before discussing the integrity of managing techniques and challenges, it is significant to understand the current situation of breaches and healthcare information disclosure risk in healthcare organizations worldwide and specifically in the Saudi Arabian context.

Data breach situations in a worldwide scenario look like a disaster for information security in the healthcare sector. Continuous cyber‐attacks are penetrating various healthcare organizations a daily worldwide. Department of Health and Human Services (HHS) of the USA released a statement and describes that HHS is targeted by attackers and notice a rapid growth in website hits [4]. The department said that attackers are trying to implement distributed denial of service attack on HHS's server and try to make various facilities unavailable of users. A research on the data breach on healthcare carried out in the period of 2009–19 was performed by an online survey journal, HIPPA. This paper reveals that the data breach on the healthcare sector is now the inferior relative to 2009 [5]. The potential attacks illustrate that data breach on the healthcare industry requires some guaranteed safeguard for securing healthcare information or electronic medical records. A study on various healthcare service providers shows that 85% of devices in medical organizations are using and running on outdated operating systems or infrastructure [6]. This kind of situation develops an open path for attackers to exploit vulnerabilities and harm the healthcare sector effectively.

Furthermore, in Saudi Arabia, cyber‐security experts believe that Saudi Arabia is a new target for cyber‐attack intruders [7]. Rapid digitalization in the healthcare sector of KSA opens a door of heaven for bad actors. It creates a disastrous situation for patients and medical organizations in the instance of health information security. All these types of statistics motivate the researchers to develop secure and much‐safeguarded information security techniques to maintain the integrity of medical record in the healthcare sector. To contribute in this context, authors examine the previous literature of the healthcare data integrity management techniques in the following heading.

**FIGURE 1:**
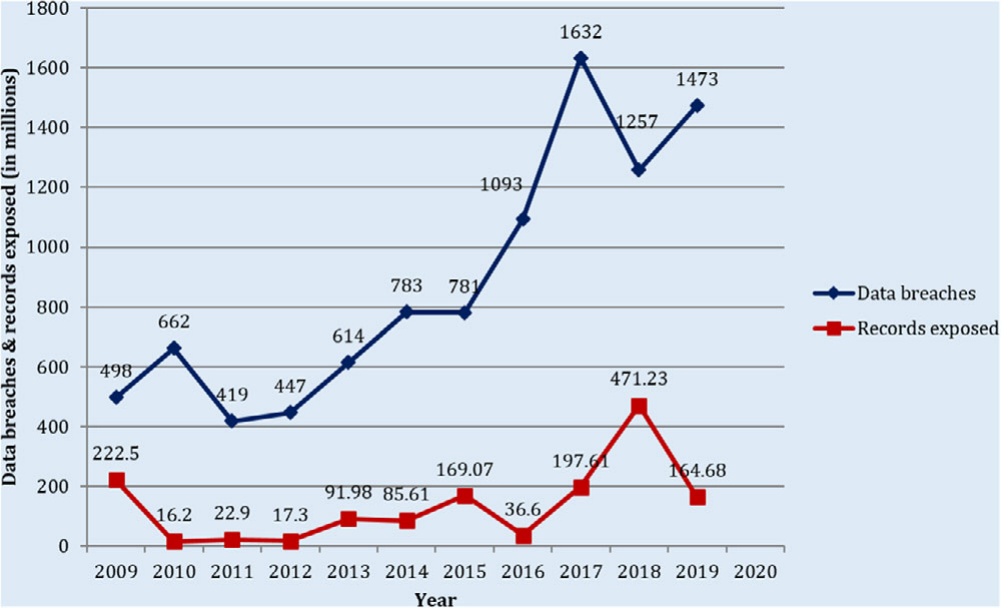
Total data breaches and records exposed graph

This research indicates that the data breach on the healthcare sector is actually in its worst state to 2009 [4]. Figure 1 depicts that adequate safeguard against malware attacks are required by healthcare sector to maintain the integrity, confidentiality and availability of data.

The HIPPA's report cites 25 of the healthcare sectors biggest data breaches in last ten years. Authors classified the attack introduced most frequently in healthcare organizations with the aid of that record. The hacking of the confidential information is the primary factor of infringements of the medical data. This should be pointed out, however, that the identification of hacking accidents is much stronger by medical organizations. The low percentage of hacking/IT attacks may be attributed to the fact that hacking events and malware attacks had not been identified in the earlier years.

**FIGURE 2:**
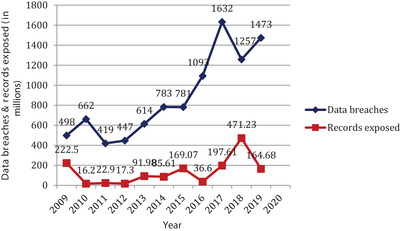
Healthcare sector breches percentage ratio

**FIGURE 3:**
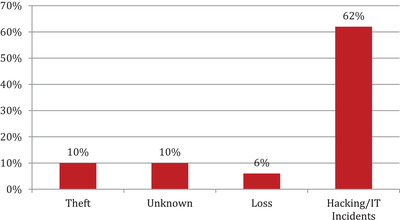
Country wise total data record stole [37]

As with hacking, the monitoring of insider infringement and notification of such violations to the Office of Civil Rights is strengthened by healthcare organizations. These accidents include employees' mistakes, incompetence and suspicious insiders' activities. Figure 2 depicts that IT incidents alone account for 62% of the largest healthcare attacks, and this is a significant ratio for any sector [4]. They require a comprehensive and fool proof entity for maintaining the data integrity, which is demonstrated by critical examination of this form of categorization. Cyber‐attacks on their network were reported by 94% of healthcare organizations [5]. Number of breaches thrice in 2018 respect to 2017, an annual healthcare sector breach analysis report shows [6]. An online news reveals that the average cost on the dark web of any healthcare record is from $1 to $1000 [7]. In 2019, by targeting their addresses 16,819, cancer patients’ records were revealed at Cancer Treatment Centers of America [8]. The American Medical Collection Agency was hacked for eight months in early May 2019, based on an online website, and 25 million patients' records were stolen at the time period. During this attack, data rated as confidential, such as the patient's credit/debit card record and prescription, was settled [9]. Figure 3 shows the country‐wise representation of the data of the total number of stolen records.

The latest data breach events happened in two major healthcare industries, LabCorp and Quest Diagnostics, the incidents settled approximately 19 million patients' records through a shared service provider [10]. The Global Healthcare Cybersecurity Market is expected to reach 27 billion USD till 2025, according to a recent research study by Global Market Insights [11]. The data breach of 10,993 patients in the American Baptist Homes of the Midwest by compromising emails and network serves was another shocking case in 2019 [12].

The statistics examined clearly reveal the patterns in attack and include a history of attacks on healthcare in past years. A crucial analysis of these attacks offers a clear condition of healthcare services data integrity. Manipulation of data often creates anxiety. The implications of uncertainty are terrifying in today's data‐driven environment. Violation of data integrity will compromise the fundamentals of infrastructure, national security, commerce, political systems and health. Alteration of data is more subtle, corrupting not only the secret in an industry's ability to secure its data, but also questioning the integrity of data from the industry. Consider the implications of attackers exploiting confidential military and government data from doctors. Exposure of highly sensitive records can be the cause of major disasters. This circumstance calls for immediate need to present the state of healthcare data integrity analysis.

## 3 RELATED WORKS

The study targeted numerous healthcare‐related SLR's to perform a systematic literature review. Most of these have addressed administrative characteristics and needs, and a few have explored different approaches to privacy and data protection. While data integrity management of healthcare is the most critical and demanding subject for modern security scientists and scholars, authors have also noticed that there is not much literature is published on the healthcare data integrity issues. However, whichever survey is available, it does provide useful information. Most scholars in their studies have explicitly concentrated on defining data integrity approaches or healthcare methodologies.

Fernández‐Alemán et al. (2013) provide a comprehensive review of existing literature to evaluate findings on electronic health record (EHR) protection and privacy schemes. The paper analysed the establishment of requirements and the perpetuation of directives relating to protection and privacy in EHR technology in recent years. More work must be done, nevertheless, to enforce these regulations and implement healthy EHR systems. Rezaeibagha et al. (2015) presented the findings of a systematic review of the existing literature on commonly implemented technological features of EHR systems in terms of protection and privacy. Pandey et al. (2020) illustrate the criticality of health information integrity concerns across the first portion of the target statistics. The second part of the article systematically reviews recent work on healthcare‐related studies of comprehensive literature and data integrity methods in the healthcare industry.

The research studies listed above provide valuable information for the healthcare sector with the help of SLRs. The authors noticed that there is a need for a SLR that consists of different strategies for data integrity and presents prospective researchers with a guide to demonstrate their research activities. The proposed research initiative explores the different data integrity management strategies discussed in top quartile research papers to reach this aim.

## 4 LITERATURE EXAMINATION

To conduct a literature analysis on this topic, we analysed the previous literature of relevant topics and fetched the proposed techniques and work done by the researcher. After the successful analysis of previous work, the authors conduct a unit analysis of selected studies for analysing the work of researchers in sub‐domains of healthcare infrastructure. Furthermore, a successful unit analysis of studies evaluates the priority of various previous data integrity techniques through a hierarchy with a fuzzy AHP technique.

### 4.1 Research objective

The biggest and most significant driver of this proposed SLR is the rapid increase in healthcare data breaches and frequent data manipulation incidents in healthcare. The second most significant driver is securing the healthcare sector. To fulfil the purpose of the proposed SLR, the authors chose following two main objectives:

Objective 1: What are the data integrity approaches that are applied worldwide in past years for managing the integrity of information and electronic records in healthcare?

Motivation: It is necessary to understand and afterward accumulate the available strategies and methodologies which have already been undertaken in this way to construct practical solutions to prevent data breach incidents. Therefore, for a comprehensive guide, this SLR aims to incorporate and extensively characterize the literature available. The SLR will therefore be an archive to be referred to by potential researchers. Also, the key reason for choosing this target was to attract the research group's attention to this critical topic.

Objective 2: Which data integrity technique is most appropriate for the KSA?

Motivation: The authors planned to include a list of prioritizations of the methods in data integrity corresponding to their need, which will benefit potential researchers. It will also allow potential researchers to prioritize past studies to pick the most appropriate solution and to consider the needs of the healthcare industry.

Objective 1 provides a descriptive analysis of previous studies published with quality journals (for making results validated). This objective provides general information on all the topmost data integrity techniques documented recently in the literature. Objective 2 is inherited by objective 1 and provides a selected technique of data integrity for the KSA and provides a systematic path to the Saudi researchers to conduct their research in electronic medical records security.

### 4.2 Methodology

In the process of conducting SLR author's main aim is including data integrity‐related quality publications. To achieve this goal, authors use various scientific databases like—Pubmed, Science Direct, IEEE Xplore and google scholar. Although, for conducting an accurate search, the following keywords were used—healthcare data integrity, electronic medical record security, healthcare information security, medical data transfer etc. with a Boolean operator AND. After all these study searching, we apply the inclusion and exclusion criteria for filtering the most relevant studies. 110 experiments were listed at the primary stage, out of which 89 accounts were recognized through examining database and 21 extra accounts were recognized through further offline sources like conference proceeding, symposium reports, books etc. The authors listed 20 related studies for performing the SLR integrity of healthcare data. The adopted inclusion criteria were defined as follows:

- The paper provided studies addressing integrity of data as a security in healthcare concern and proposing some quantitative solutions.
- SLR contains articles that discuss the issue of healthcare reputation using a particular approach.
- SLR contains only the studies reported in Q1 and Q2 journals (for reliability and completeness of results).
- SLR provides research that has some definitive evidence on healthcare credibility issues.

For excluding criteria were defined as follows:

- Exclude articles which did not apply the conditions of the request and the examination intention.
- Exclude articles that addressed data integrity but not from the viewpoint of healthcare.
- Exclude records that are not accurate and definitive to support the healthcare problem of data integrity.

**FIGURE 4:**
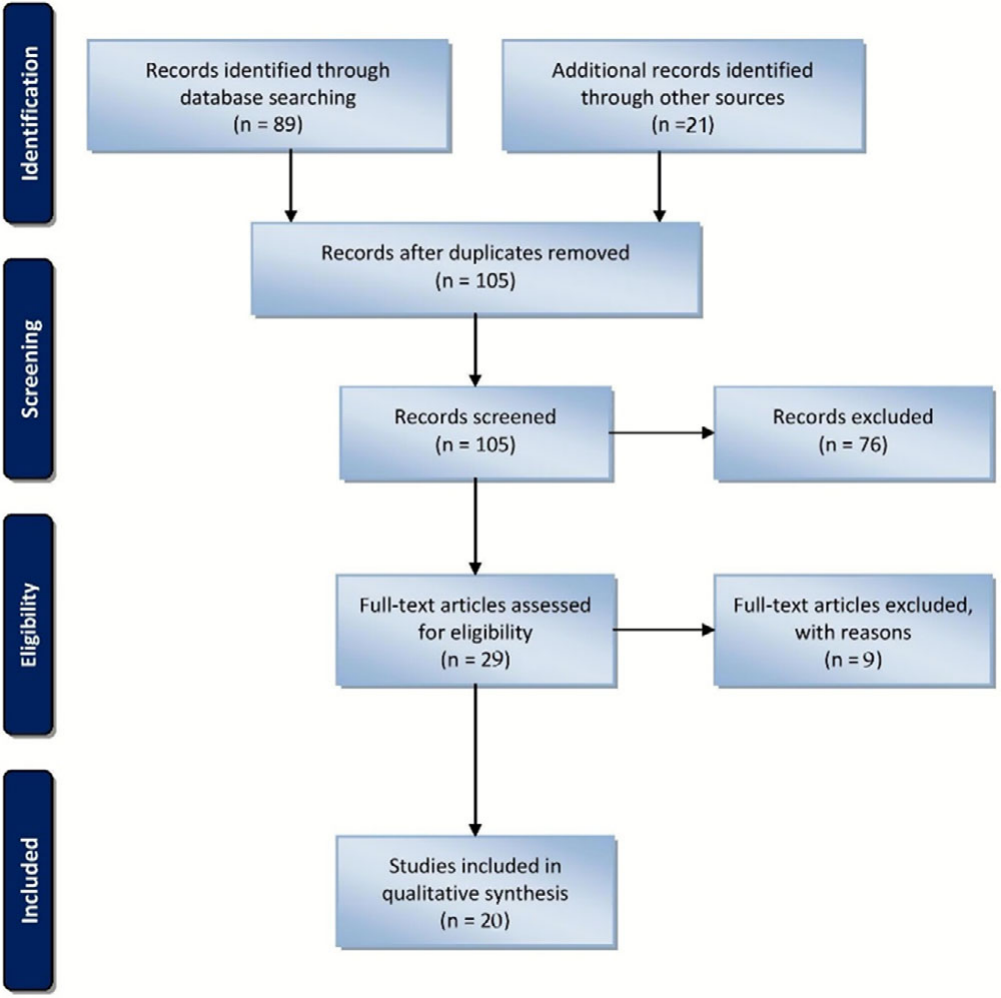
PRISMA flow diagram for paper selection

As mentioned in Figure 4, the researchers excluded the articles in the screening and eligibility process based on their analysis.

In the second point, after screening the complete report, the authors omitted the articles. In this step, 76 articles were omitted, which were not appropriate for SLR. Furthermore, 9 more articles were omitted in eligibility checking step after full text articles review. Preferred reporting items for systematic literature review and meta‐analysis 2009 flow diagram are used by researchers to illustrate the paper selection criteria. This approach has been implemented by ref. [13] and lays down guidelines for the development of Systematic studies and meta‐examination. Table 1 explains the studies in tabular format and their corresponding percentages.

**TABLE 1:** Literature search figures

**Data repository**	**Relevant papers**	**Not‐relevant papers**	**Total**	**Relevant percentage (%)**
PubMed	4	21	25	16
Science direct	5	17	22	22.72
Google scholar	5	15	20	25
IEEE Xplorar	4	18	22	18.18
Cite‐seeker	2	14	16	12.5
Total	20	85	105	19.04

To fully comprehend the search process, the various search figures from various digital repositories have been taken. The goal of the researchers is to identify the existing data integrity strategies used by healthcare organizations through a comprehensive analysis and emphasize the criticality of healthcare data integrity issues. This was accomplished by gathering data on different statistics of the breaches.

### 4.3 Exploratory analysis of results

See Table 2 for an overview of the included research. The table displays the key material of the research findings and their respective method of data integrity. It was noticed during the review that certain papers had mentioned the issues of ethics and healthcare in their literature. Therefore the authors included all the articles for this SLR for a much more thorough analysis. The summary of the various methods of data integrity used during earlier studies is summarized below.

**TABLE 2:** Exploratory analysis of studies

**Author**	**Study description**	**Technique for data integrity**
William J. Gordon et al. (2018) [14]	The study provides descriptive information of how to facilitate the block chain approach in healthcare sector. The paper also discusses about the challenges that are associated with blockchain in order to provide a secure communication.	Blockchain
James Brogan et al. (2018) [15]	The study provides distributed Ledger technologies in advancing electronic health information's. The paper provides a cost‐effective and novel approach for the healthcare organization.	Masked authenticated messaging extension
Peng Zhang et al. (2018) [16]	The paper provides a blockchain‐based architecture FHIR‐chain for securing Medicare.	Blockchain
Christian Esposito et al. (2016) [17]	The study uses cloud storage environment for data available in healthcare organizations and for patients. Authors also use blockchain approach for secure lab report transaction and communication.	Blockchain
Prosanta Gope et al. (2015) [18]	The study uses body sensor network approach for facilitating secure and integrity managed architecture of IoT in healthcare.	Secure‐BSN
P. Vimalachandran et al. (2017) [19]	Authors proposed authorization based model for Australian healthcare services.	Authentication
M. ELHOSENY et al. (2018) [20]	The study provides a stenographic technique with hybrid encryption mechanism for securing health records and images.	Encryption
EntaoLuo et al. (2018) [21]	The study provides a secure sharing based data transfer in IoT environment for data security of healthcare organization.	Slepian‐ Wolf‐coding‐based secret sharing (SW‐SSS)
Moshaddique Al Ameen et al. (2010) [22]	The paper discusses about the challenges and issues associated with the wireless sensors in healthcare sector.	‐
Gunasekaran Manogaran et al. (2017) [23]	Authors give a secure organizational IoT based model for storing and processing wearable sensor data in medical services.	Secure Cloud
Benjamin Fabiana et al. (2014) [24]	The study provides inter organizational data transfer security through various security attributes. The paper provides the architecture for secure data transfer from one organization to another.	Secure Cloud
Jinyuan Sun et al. (2011) [25]	The paper provides a secure health record system for patient privacy based on cryptographic techniques and IoT environment of healthcare industry.	Cryptography
Abdullah Al Omar et al. (2017) [26]	The study presents a data management system for healthcare services to facilitate patients through blockchain technology.	Blockchain
Sue Bowmanet et al. (2013) [27]	The study highlights the current challenges and other error causes in healthcare data integrity in healthcare organization. The paper provides a review on current HER system of healthcare.	‐
Anastasia Theodouli et al. (2018) [28]	The study presents mechanism for facilitating blockchain technology for providing auditable and sharable data in healthcare organization.	Blockchain
Zarour et al. (2020) [29]	The study used hybrid fuzzy based methodology for evaluating the impact of different blockchain technology models in a healthcare perspective.	Blockchain
Karim Abouelmehdi et al. (2018) [30]	In this study, the authors have discussed about the challenges and survey the current situation of healthcare big data.	‐
Anam Sajid et al. (2016) [31]	The study presents review on healthcare medical data security for providing privacy to the patients. Paper also discusses about the currently used techniques and approaches in healthcare system.	‐
Brihat Sharma et al. (2018) [32]	The study proposes a model, the Merkle tree‐based approach to secure the integrity of health records. The software model closely refers to the Blockchain technology.	Merkle tree‐based approach
Katharine Gammon (2018) [33]	The article illustrates the blockchain application in healthcare sector in various domains.	‐

#### 4.3.1 Blockchain approach

The blockchain technology approach has been used by many studies as a critical element in their studies to securely manage health care data, including:

- Study suggested by William J. Gordon et al. explores how the blockchain solution in an organization of healthcare sector is facilitated [14]. The research article addresses the problems and concerns and presents a new model for promoting the implementation of blockchain in health related facilities.
- Peng Zhang et al. published a model addressing the security of scientific data and providing FHIR‐chain architecture based on blockchain [16].
- Abdullah Al Omar et al. published an article addressing blockchain as health related storage [26].
- Anastasia et al. designed an innovative blockchain method for simplifying hospital related data auditable, sharable, as well as, securely usable [28].
- Zarour et al. used a hybrid fuzzy based methodology for assessing the effect of different blockchain technology models and delivers an original awareness and track to the future scientists [29].

#### 4.3.2 Masked authenticated messaging extension

James Brogan et al. published an article on the security enhancement of patient data in connected medical devices via the masked identity verification message extension module [15]. The authors developed a relationship in their paper between IOTA, as well as, masked authentication messaging extensions and resolved the problems that wearable technology face. The IOTA strategy was developed to be compact and flexible to deliver as the foundation for secure data connectivity among the Internet‐of‐Things (IoT) systems. It distinguishes itself from conventional blockchain‐based distributed ledger procedures by acknowledging two major specific problems: latency and expenses. For potential researchers, the methodology which is used in this article is very helpful.

- Secure‐body sensor network (BSN): Prosanta Gope et al. published an article on the strategy of the BSN in the IoT setting of healthcare. BSN method is the core technology in the IoT healthcare setting, where a patient is tracked using tiny compact body sensors [18]. For safe IoT communication with healthcare providers, the paper offers a stable, as well as, integrity‐manageable BSN method.
- Authentication: P. Vimala et al. suggested a step towards identity verification in Australian healthcare services [19]. The identity verification step provides patients with a realistic monitoring of data, and can monitor data access with the help of this novel strategy.
- Encryption: M. ELHOSENY et al. published an article on healthcare image protection and patient records in image format, as well as, other forms. Steganography and a hybrid encryption system are given in the paper to protect healthcare data. For good outcomes in the future the strategy needs more research [20].
- Wolf‐coding‐based secret sharing: Entao Luo et al. establish comprehensive, stable IoT communication and information sharing among two IoT devices [21]. The researchers used a Wolf‐based sharing approach to safeguard the healthcare IoT setting.
- Secure Cloud: Gunasekaran Manogaran et al. published a model dealing with the big data situation in the latest healthcare industry and offering a stable cloud solution for the management of broad healthcare data [23]. Benjamin Fabiana et al. moreover discussed the inter‐organizational information exchange through safe cloud tactic.
- Merkle Tree‐based approach: Brihat Sharma et al. published a study offering a strategy that is used in the healthcare services to secure data transmission and communication [32]. The proposed solution mimics blockchain solution and aims to provide such a better and more reliable data sharing and statement setting.

### 4.4 Unit analysis

In this study the unit analysis is significant step of a methodical analysis wherein the researchers identify and classify the revisions as per their respective healthcare field of study. For case, if a research offers a complete integrity‐managed framework for the entire system then the sub‐classification specified in this SLR is “entire healthcare system” and if a research work just encompasses the safe communication among IoT procedures then the subcategory of such an area is data transfer. The numerous researches covering various facets of the healthcare platform for data integrity protection are outlined in Table 3.

**TABLE 3:** Unit analysis

**Authors**	**Data soundness**	**Data auditability**	**Privacy‐preserving**	**Data honesty**	**Data backup**
William J. Gordon et al. (2018)		√	√		
James Brogan et al. (2018)		√			
Peng Zhang et al. (2018)			√		
Christian Esposito et al. (2016)		√	√	√	
Prosanta Gope et al. (2015)	√				
P. Vimalachandran et al.	√				
M. ELHOSENY et al. (2018)					√
Entao Luo et al. (2018)			√		
Moshaddique Al Ameen et al. (2010)			√		√
Gunasekaran Manogaran et al.			√	√	
Benjamin Fabiana et al. (2014)		√			
Jinyuan Sun et al. (2011)					√
Abdullah Al Omar et al. (2017)				√	
Sue Bowman et al. (2013)		√		√	
Anastasia Theodouli et al.		√	√		
Xueping Liang et al. (2017)		√		√	
Karim Abouelmehdi et al. (2018)	√		√		
Anam Sajid et al. (2016)		√		√	
Brihat Sharma et al. (2018)					√
Katharine Gammon (2018)					

Table 3 summarizes the latest published studies that concentrated on various areas of the health industry. In contrast to the other facets of healthcare facilities, the table demonstrates that improving healthcare record integrity needs more importance. A good integrity‐managed process is also needed via multiple data integrity management strategies for the entire healthcare system.

### 4.5 Scientometric analysis

The researchers conducted the in the third stage, the researches performed the Scientometric analysis to understand which methodology of data integrity must be given greater research interest. Scientific research is a qualitative examination of studies. This description was first established by ref. [33]. The quantitative and qualitative outcomes of the studies are summarized in Table 4 by the authors, journal indexing, ranking, group and quartile. As per their Indexed category, the quartile area contains all the types the journals provided.

**TABLE 4:** Scientometric analysis

**Authors**	**Journal**	**Quartile**	**Category**
William J. Gordon et al. (2018)	Computational and Structural Biotechnology Journal	Q1	Computer science application
James Brogan et al. (2018)	Computational and Structural Biotechnology Journal	Q1	Computer science application
Peng Zhang et al. (2018)	Computational and Structural Biotechnology Journal	Q1	Computer science application
Christian Esposito et al. (2016)	IEEE Cloud Computing	Q1	Computer science (miscellaneous)
Prosanta Gope et al. (2015)	IEEE SENSORS JOURNAL	Q1	Electrical and electronic engineering
P. Vimala Chandran et al.	2017 International Conférence on Orange Technologies (ICOT)	‐	‐
M. ELHOSENY et al. (2018)	IEEE Access	Q1	Engineering (miscellaneous)
Entao Luo et al. (2018)	IEEE Communications Magazine	Q1	Computer networks and communications
Moshaddique Al Ameen et al. (2010)	Journal of Medical Systems	Q2	Health informatics
Gunasekaran Manogaran et al.	Thames L., Schaefer D. (eds) Cybersecurity for Industry 4.0. Springer Series in Advanced Manufacturing	‐	‐
Benjamin Fabiana et al. (2014)	Information Systems	Q1	Information system
Jinyuan Sun et al. (2011)	2011 31st International Conference on Distributed Computing Systems	‐	‐
Abdullah Al Omar et al. (2017)	International Conference on Security, Privacy and Anonymity in Computation, Communication and Storage	‐	‐
Sue Bowmanet al. (2013)	Perspective Health Information Managing	Q2	Medicine (miscellaneous)
Anastasia Theodouli et al.	2018 17th IEEE International Conference On Trust, Security And Privacy In Computing And Communications/ 12th IEEE International Conference On Big Data Science And Engineering(TrustCom/ BigDataSE)	‐	‐
Xueping Liang et al. (2017)	2017 IEEE 28th Annual International Symposium on Personal, Indoor, and Mobile Radio Communications (PIMRC)	‐	‐
Karim Abouelmehdi et al. (2018)	Journal of Big Data	Q1	Information system and management
Anam Sajid et al. (2016)	Journal of Medical Systems	Q2	Health information management
Brihat Sharma et al. (2018)	2018 9th IEEE Annual Ubiquitous Computing, Electronics & Mobile Communication Conference (UEMCON)	‐	‐
Katharine Gammon (2018)	Nature Medicine	Q1	Medicine (miscellaneous)

Table 4 explicitly indicates that the highest number of publications is in the field of computer science. In the computer science group, a total of 5 articles published are accessible. The groups of informatics and health knowledge have two, two articles, respectively. Medicine (miscellaneous) has a group of two publications. There are also two publications for the engineering group. All these figures indicate that the focus of research in computer science to solve the data integrity issue in the healthcare sector is rising comparatively strong.

Except the Journal of Computational and Structural Biotechnology (CSB), only one paper was published in all the journals. The CSB journal published three papers on the integrity of data. Paper quartiles explicitly demonstrate that the standard of research work is quite useful in data integrity strategies of healthcare, as there is a lack of research study in this field. It is specifically encouraged to implement and perform high‐quality research continually to achieve the exploitation of free data processing in medical services.

### 4.6 Ranking/priority analysis

To quickly grasp the previous situation, the above overview of studies assorts in the previous research of data integrity strategies in the health care system into different criteria. The researchers introduced a methodology for the ranking study using an efficient Fuzzy‐AHP to prioritize the techniques such as data integrity and presented the research community with the top placed method.

**FIGURE 5:**
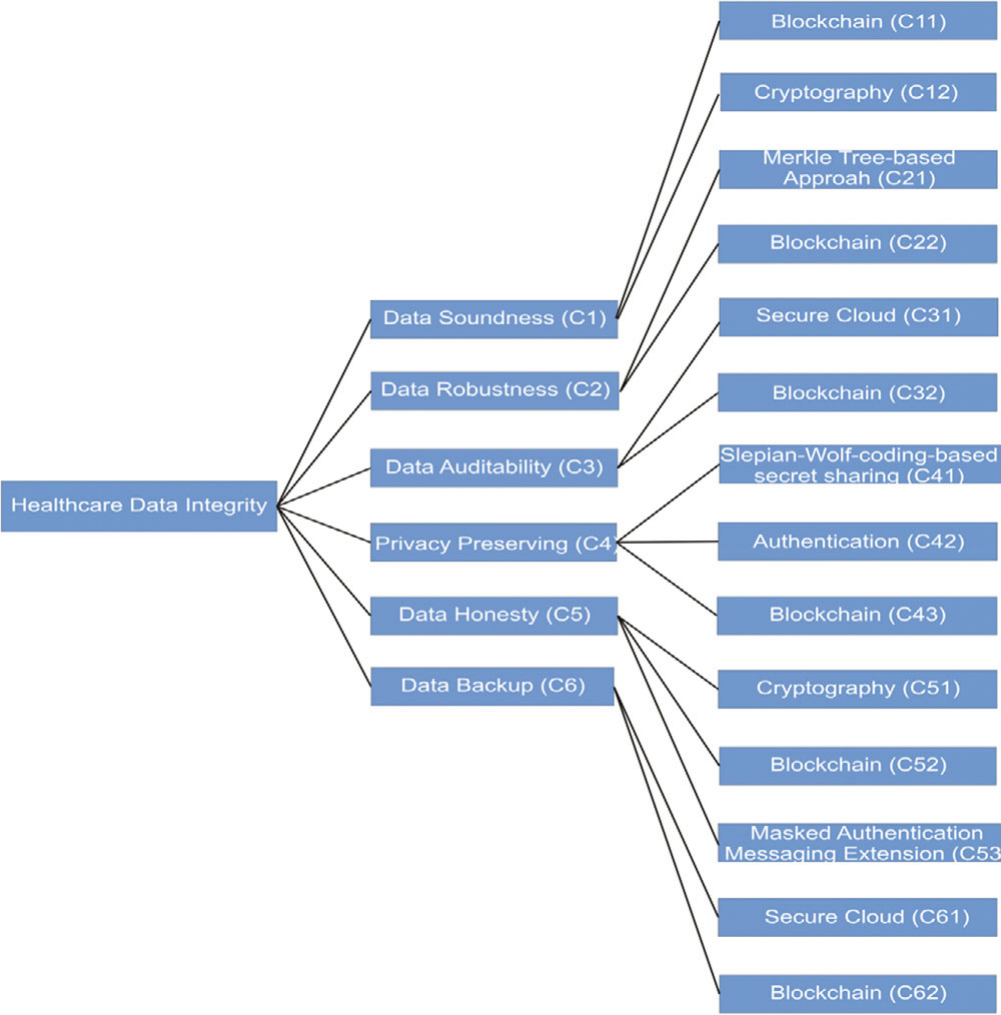
Data integrity approaches hierarchy and their sub‐fields of healthcare

The authors also developed a hierarchy of data integrity methods spanning multiple sub‐levels of the system to analyse the prior studies and implement AHP. The hierarchy of integrity strategies in various healthcare contexts is shown in Figure 5.

The above hierarchy defines different methods of data integrity that are used in various sub‐fields of the system. To determine the priority of the data integrity methods, the authors applied the Fuzzy‐AHP procedure.

Fuzzy‐AHP is useful in eliciting precise values/facts during making decisions [34]. A commonly applied priority evaluation tool is Fuzzy‐AHP. The researchers of this work have previously used this procedure [34] to perform the Fuzzy‐AHP approach and have gathered data from 75 experts from various areas. This approach focuses on giving the prioritized data procedure in healthcare with the aid of feedback from experts. With the help of Figure 5 data integrity procedures used 5.We have prepared the aggregated fuzzy comparison metrics with the assistance of [34, 35].

The fuzzy based pair‐wise comparison matrix for data backup at level‐1depicts in Table 5 that contains data soundness, data robustness, data auditability, privacy‐preserving, data honesty and data backup. The fuzzy based pair‐wise comparison matrix for data backup at level‐2 of data soundness depicts on Table 6. Level‐2 of data soundness consists of Blockchain and cryptography techniques. Table 7 depicts the fuzzy based pair‐wise comparison matrix for data backup at level‐2 of Data robustness. Level‐2 of data robustness consists of Markle Tree‐based procedure and blockchain. The fuzzy based pair‐wise comparison matrix for of data auditability at level‐2 is depicted in Table 8. Level‐2 of data auditability consists of secure cloud and Blockchain techniques. The fuzzy based pair‐wise comparison matrix for privacy‐preserving of level‐2 has been shown in Table 9. Level‐2 for privacy preserving contains Slepian Wolf coding based sharing, authentication and blockchain. The fuzzy based pair‐wise comparison matrix for data honesty of level‐2 is depicted in Table 10 that includes data honesty and contains cryptography, Blockchain and masked authentication messaging extension. The fuzzy based pair‐wise comparison matrix for data backup of level‐2 that includes Blockchain and Secure cloud technique is depicted in Table 11. The authors followed [34] processes to test the defuzzify and RC values of the matrix. This study used the alpha cut procedure [34] for the defuzzification process. The defuzzified pair‐wise comparison matrix is depicted by Table 12. Tables 13, –18 also depict the combined pair‐wise comparison matrix for all groups. Finally, in Table 19, dependent weights were seen via the hierarchy.

**TABLE 5:** Fuzzy based pair‐wise comparison matrix

	**(C1)**	**(C2)**	**(C3)**	**(C4)**	**(C5)**	**(C6)**
(C1)	1.00, 1.00, 1.00	1.35, 1.82, 2.39	1.41, 1.97, 2.48	0.31, 0.44, 0.63	0.87, 0.90, 0.95	0.23, 0.29, 0.42
(C2)	‐	1.00, 1.00, 1.00	0.85, 1.11, 1.45	0.55, 0.89, 1.37	0.79, 0.88, 1.02	0.25, 0.33, 0.50
(C3)	‐	‐	1.00, 1.00, 1.00	2.04, 3.16, 4.23	0.26, 0.36, 0.59	0.69, 1.00, 1.51
(C4)	‐	‐	‐	1.00, 1.00, 1.00	0.36, 0.52, 0.96	0.36, 0.52, 0.80
(C5)	‐	‐	‐	‐	1.00, 1.00, 1.00	0.89, 1.14, 1.39
(C6)	‐	‐	‐	‐	‐	1.00, 1.00, 1.00

**TABLE 6:** Fuzzy based combined pair‐wise comparison matrix for data soundness at level‐2

	**(C11)**	**(C12)**
(C11)	1.00, 1.00, 1.00	0.49, 0.70, 0.93
(C12)	‐	1.00, 1.00, 1.00

**TABLE 7:** Fuzzy based combined pair‐wise comparison matrix for data robustness at level‐2

	**(C21)**	**(C22)**
(C21)	1.00, 1.00, 1.00	0.40, 0.54, 0.78
(C22)	‐	1.00, 1.00, 1.00

**TABLE 8:** Fuzzy based combined pair‐wise comparison matrix for data auditability at level‐2

	**(C31)**	**(C32)**
(C31)	1.00, 1.00, 1.00	0.80, 1.23, 1.78
(C32)	‐	1.00, 1.00, 1.00

**TABLE 9:** Fuzzy based combined pair‐wise comparison matrix for privacy at level‐2

	**(C41)**	**(C42)**	**(C43)**
(C41)	1.00, 1.00, 1.00	0.48, 0.67, 0.89	0.59, 0.70, 0.90
(C42)	‐	1.00, 1.00, 1.00	0.27, 0.38, 0.63
(C43)	‐	‐	1.00, 1.00, 1.00

**TABLE 10:** Fuzzy based combined pair‐wise comparison matrix for data honesty at level‐2

	**(C51)**	**(C52)**	**(C53)**
(C51)	1.00, 1.00, 1.00	0.55, 0.58, 0.66	0.63, 0.91, 1.34
(C52)	‐	1.00, 1.00, 1.00	0.42, 0.63, 0.96
(C53)	‐	‐	1.00, 1.00, 1.00

**TABLE 11:** Fuzzy based combined pair‐wise comparison matrix for data backup at level 2

	**(C61)**	**(C62)**
(C61)	1.00, 1.00, 1.00	0.38, 0.54, 0.83
(C62)	‐	1.00, 1.00, 1.00

**TABLE 12:** Combined pair‐wise comparison matrix and local weights at level 1

	**(C1)**	**(C2)**	**(C3)**	**(C4)**	**(C5)**	**(C6)**	**Weights**
(C1)	1.00	1.84	1.95	0.45	0.90	0.30	0.146
(C2)	0.54	1.00	1.13	0.93	0.89	0.36	0.114
(C3)	0.51	0.88	1.00	3.15	0.39	1.05	0.160
(C4)	0.94	1.07	2.51	1.00	0.59	0.55	0.131
(C5)	1.80	1.11	1.67	0.317	1.00	1.14	0.208
(C6)	0.87	2.20	1.10	3.25	2.77	1.00	0.241
							C.R. = 0.00932

**TABLE 13:** Combined pair‐wise comparison matrix for data soundness at level‐two

	**(C11)**	**(C12)**	**Weights**
(C11)	1.00	0.71	0.415
(C12)	1.41	1.00	0.585
C.R. = 0.000			

**TABLE 14:** Combined pair‐wise comparison matrix for Data robustness at level‐two

	**(C21)**	**(C22)**	**Weights**
(C21)	1.00	0.57	0.363
(C22)	1.74	1.00	0.637
			C.R. = 0.000

**TABLE 15:** Combined pair‐wise comparison matrix for data auditability at level two

	**(C31)**	**(C32)**	**Weights**
(C31)	1.00	1.26	0.558
(C32)	0.79	1.00	0.442
			C.R. = 0.000

**TABLE 16:** Combined pair‐wise comparison matrix for privacy preserving at level two

	**(C41)**	**(C42)**	**(C43)**	**Weights**
(C41)	1.00	0.66	0.73	0.251
(C42)	1.50	1.00	0.42	0.275
(C43)	1.36	2.37	1.00	0.474
CR = 0.002544				

**TABLE 17:** Combined pair‐wise comparison matrix for data honesty at level two

	**(C51)**	**(C52)**	**(C53)**	**Weights**
(C51)	1.00	0.59	1.26	0.301
(C52)	1.67	1.00	0.66	0.345
(C53)	0.78	1.50	1.00	0.354
C.R. = 0.00759				

**TABLE 18:** Combined pair‐wise comparison matrix for data backup at level two

	**(C61)**	**(C62)**	**Weights**
(C61)	1.00	0.58	0.367
(C62)	1.72	1.00	0.633
C.R. = 0.000			

**TABLE 19:** Overall weights and ranking of methods

1st level methods	Local weights of first level	Second level methods	Local weights of second level	Overall weights	Percentage	Overall ranks
(C1)	0.146	(C11)	0.415	0.06059	6.059 %	11
		(C12)	0.585	0.08541	8.541 %	4
(C2)	0.114	(C21)	0.363	0.04138	4.138 %	12
		(C22)	0.637	0.07262	7.262 %	6
		(C31)	0.558	0.08928	8.928 %	2
(C3)	0.160	(C32)	0.442	0.07072	7.072 %	8
		(C41)	0.251	0.03228	3.228 %	14
		(C42)	0.275	0.03603	3.603 %	13
(C4)	0.131	(C43)	0.474	0.06209	6.209 %	10
		(C51)	0.301	0.06261	6.261 %	9
(C5)	0.208	(C52)	0.345	0.07176	7.176 %	7
		(C53)	0.354	0.07363	7.363 %	5
(C6)	0.241	(C61)	0.367	0.08845	8.845 %	3
		(C62)	0.633	0.15255	15.255 %	1

**FIGURE 6:**
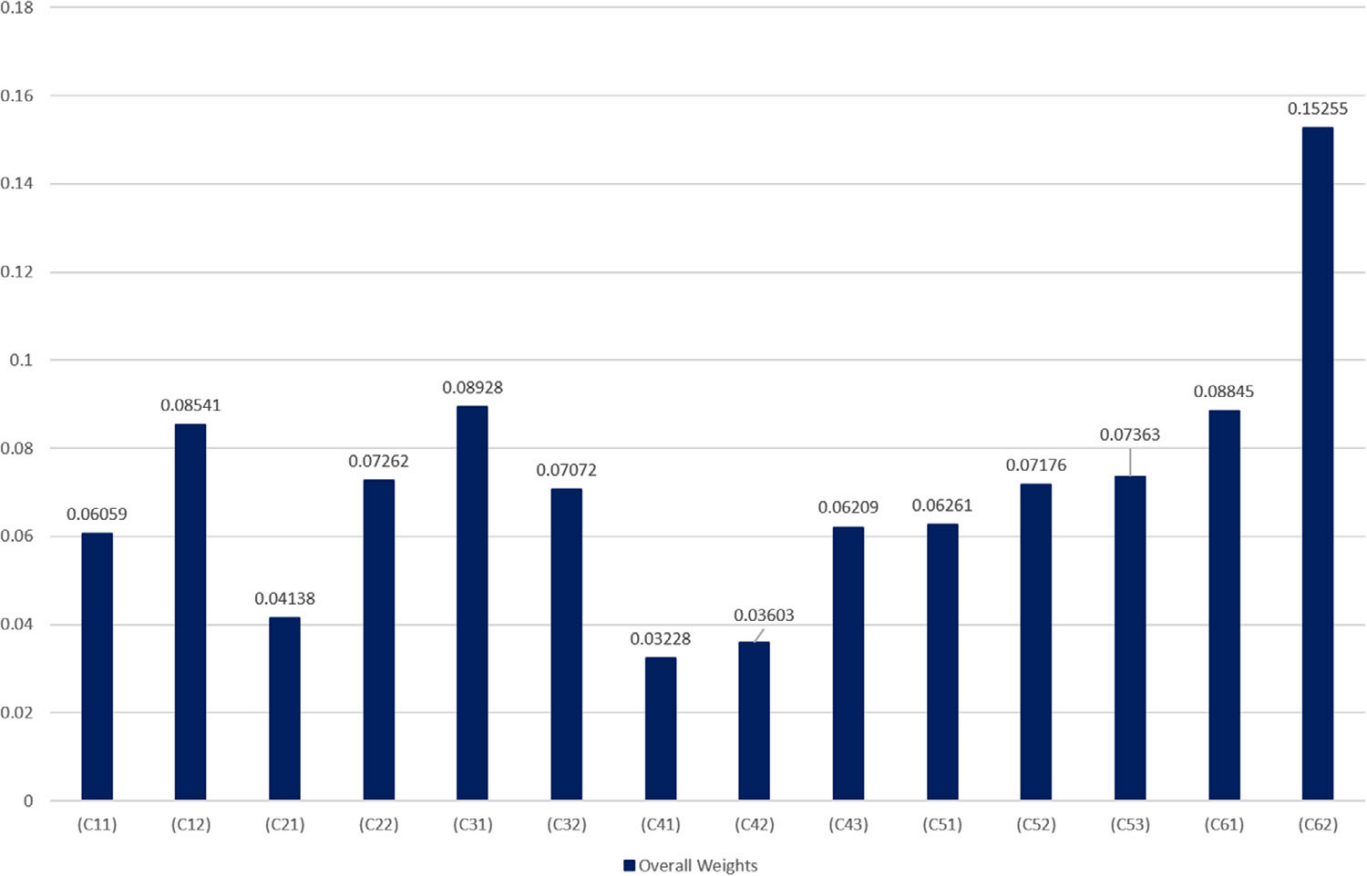
Graphical representation of global weights and ranking of data integrity approaches

Table 19 and Figure 6 summarize the outcomes acquired after the estimation of data integrity measures by the fuzzy‐AHP procedure. Based on priority ranking blockchain technology gained highest rank, the table demonstrates that of all the techniques. The results based on the Fuzzy‐AHP procedure corroborate that the investigators must concentrate on the blockchain technique for good solutions to maintain the data integrity. The researchers have investigated the blockchain issues in healthcare for more knowledge and explanation. Also, previous blockchain researches carried out in the sense of these difficulties have also been addressed. Such classification will provide abundantly clear details on the latest blockchain technique in healthcare paradigm for data transparency.

## 5 CONCLUSION

This SLR offers an overview of the current situation for healthcare data integrity by attack statistical data. Also, this research deals with previous studies of data integrity strategies to clarity of the working environment in the healthcare sector to manage data integrity. The results of this SLR strongly indicate that the healthcare sector needs a new and more robust data integrity approach. The first segment of this SLR illustrates the criticality of healthcare organizations' data integrity problems. In the second section (Review part), prospective researchers are invited to adopt and inspire data integrity studies. This SLR can be important for readers with the aid of preference evaluation. The rankings evaluate the importance of previously implemented data integrity strategies in health and identify them through the Fuzzy‐AHP methodology, which will provide a path for potential researchers relating to data integrity approaches and practices. This paper used statistical procedure, to describe the challenges and consequences. Two distinct goals were used to direct the fundamental analysis outlined in the discussion section. The first goal was to offer a simple and straightforward review using different analysis methods of earlier studies. Secondly, the data integrity strategies should be enumerated in all previously mentioned methods. Such a database will be a resource for scientists and professionals who are both investigating potential solutions to the issue of data integrity protection and implement the most prioritized technologies for collecting knowledge in the healthcare sector. The numerous studies examined and datasets accessible to authors are limited. Their results are not available. While the researchers accessed several databases, there are undoubtedly studies and datasets that cannot be included in the SLR profile.
